# Supplementary material for: Association of Insulin Resistance with Vascular Ageing in a General Caucasian Population: An EVA Study
Source: J Clin Med. 2021 Dec 8;10(24):5748. doi: 10.3390/jcm10245748 (PMC8707603; doi:10.3390/jcm10245748)
Supplement: Supplementary file 1 [file jcm-10-05748-s001.zip › jcm-1498522-supplementary.pdf]

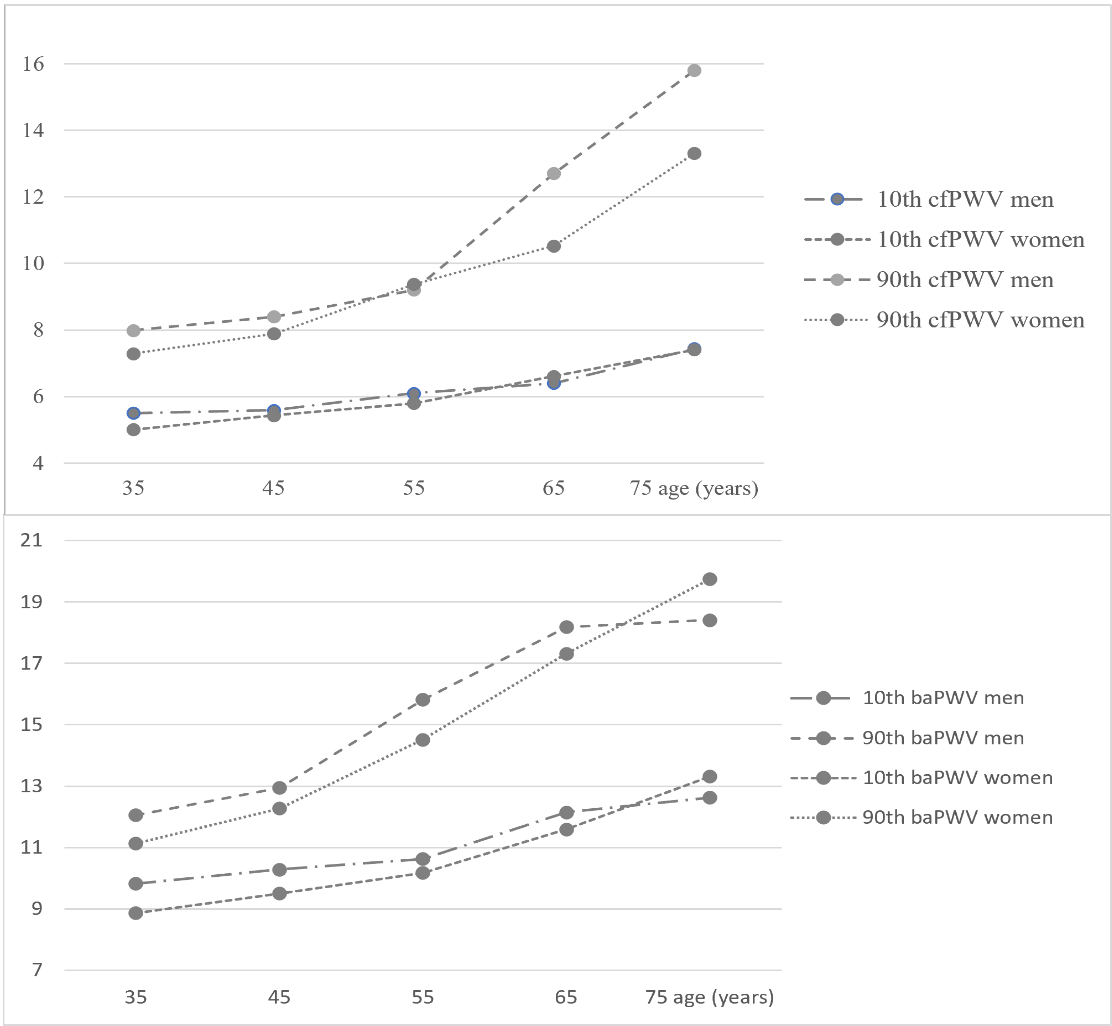

Figure S1. Age-specific 10th and 90th percentile for carotid to femoral pulse wave velocity and for braquial to ankle pulse wave velocity in participants without prevalent cardiovascular disease. cfPWV carotid to femoral aortic pulse wave velocity, baPWV brachial to ankle pulse wave velocity.

Table S1. The correlation between insulin resistance indexes and arterial stiffness parameters globally and by sex.

	cfPWV, m/sec	baPWV, m/sec
Global		
HOMA-IR	0.358**	0.355**
TyG index	0.428**	0.436**
Triglyceride/HDL-C ratio	0.258**	0.255**
LAP index	0.406**	0.386**
WHt ratio	0.471**	0.462**
VA index	0.251**	0.255**
Men		
HOMA-IR	0.369**	0.372**
TyG index	0.415**	0.422**
Triglyceride/HDL-C ratio	0.120	0.126*
LAP index	0.331**	0.292**
WHt ratio	0.511**	0.538**
VA index	0.163*	0.162*
Women		
HOMA-IR	0.304**	0.320**

	cfPWV, m/sec	baPWV, m/sec
TyG index	0.340**	0.427**
Triglyceride/HDL-C ratio	0.305**	0.300**
LAP index	0.425**	0.421**
WHt ratio	0.416**	0.389**
VA index	0.312**	0.315**

cfPWV carotid to femoral aortic pulse wave velocity, baPWV brachial to ankle aortic pulse wave velocity, HOMA-IR homeostatic model assessment of insulin resistance, TyG index triglyeride and glucose index, Triglyceride/HDL-C ratio triglyceride to high-density lipoprotein cholesterol ratio, LAP index lipid accumulation product index, WHt ratio waist-to-height ratio, VA index visceral adiposity index.

* p value <0.05

** p value <0.001

Table S2. *The correlation between insulin resistance indexes and arterial stiffness parameters globally adjusted for age and sex.*

	cfPWV, m/sec	baPWV, m/sec
Global		
HOMA-IR	0.151*	0.160*
TyG index	0.156*	0.183*
Triglyceride/HDL-C ratio	0.138*	0.198**
LAP index	0.209**	0.179**
WHt ratio	0.175*	0.134*
VA index	0.150*	0.196**

cfPWV carotid to femoral aortic pulse wave velocity, baPWV brachial to ankle aortic pulse wave velocity, HOMA-IR homeostatic model assessment of insulin resistance, TyG index triglyeride and glucose index, Triglyceride/HDL-C ratio triglyceride to high-density lipoprotein cholesterol ratio, LAP index lipid accumulation product index, WHt ratio waist-to-height ratio, VA index visceral adiposity index.

* p value <0.05. ** p value <0.001.

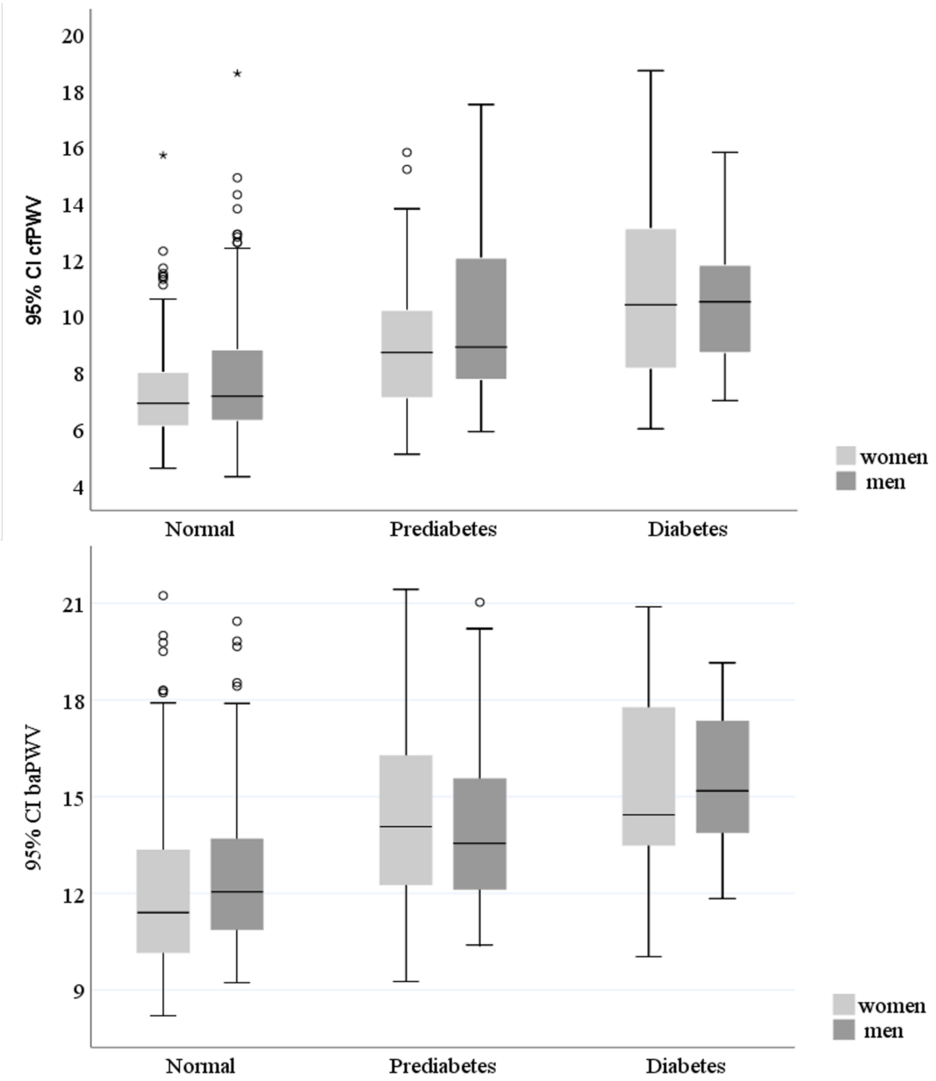

Figure S2. Values according to glucose metabolism by sex of the two measures of arterial stiffness used. The boxes contain 50% of the data; the horizontal lines indicate the values of the medians and the circles the extreme values. cfPWV femoral carotid pulse wave velocity, btPWV braquial ankle pulse wave velocity.

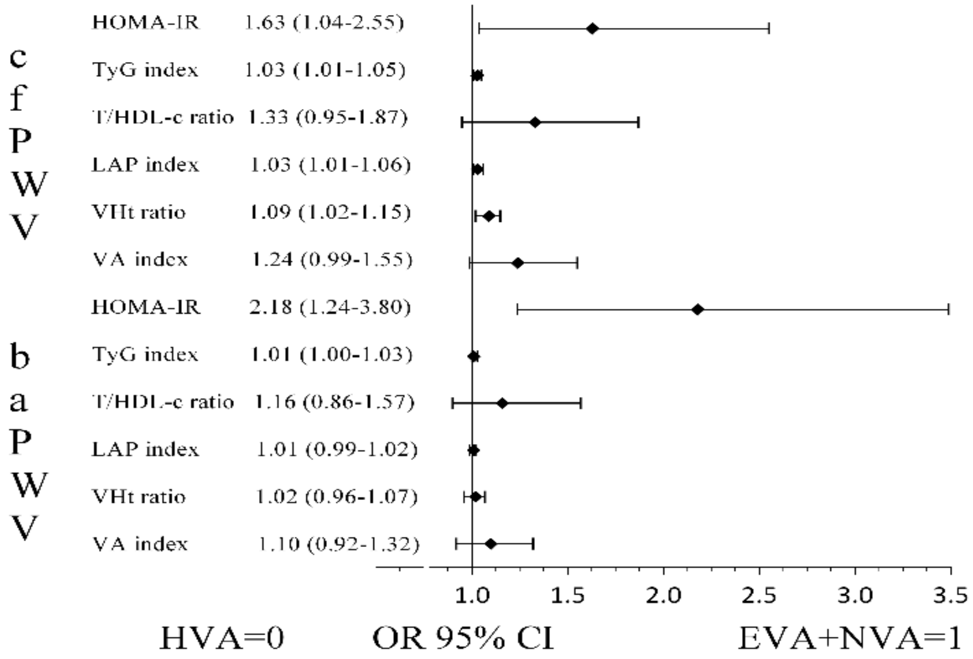

Figure S3. Odds ratio of determinants of HVA frente NVA + EVA with insulin resistance indexes in adults ages 35–75 years. Adjusted for age, sex, antihypertensive drugs, lipid-lowering and hypoglycemic drugs.

Abbreviations: EVA early vascular aging, HVA healthy vascular aging, NVA normal vascular aging, cfPWV carotid to femoral pulse wave velocity, baPWV brachial to ankle pulse wave velocity, HOMA-IR homeostatic model assessment of insulin resistance, TyG index triglyeride and glucose index, T/HDL-c ratio triglyceride to high-density lipoprotein cholesterol ratio, LAP index lipid accumulation product index, WHt ratio waist-to-height ratio, VA index visceral adiposity index.

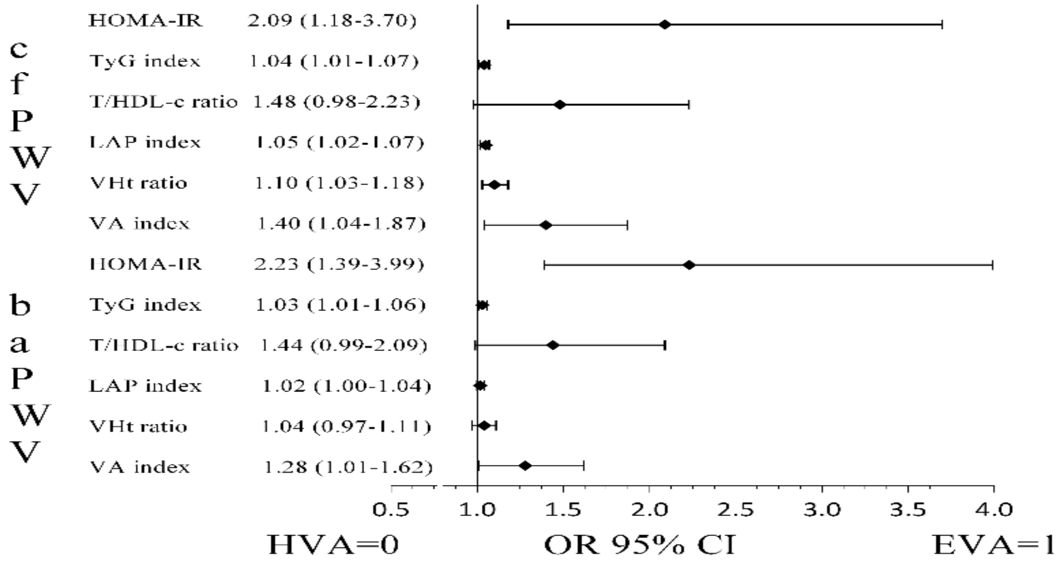

Figure S4. Odds ratio of determinants of HVA frente a EVA with insulin resistance indexes in adults ages 35–75 years. Adjusted for age, sex, antihypertensive drugs, lipid-lowering and hypoglycemic drugs.Abbreviations: EVA early vascular aging, HVA healthy vascular aging, NVA normal vascular aging, cfPWV carotid to femoral pulse wave velocity, baPWV brachial to ankle pulse wave velocity, HOMA-IR homeostatic model assessment of insulin resistance, TyG index triglyeride and glucose index, T/HDL-c ratio triglyceride to high-density lipoprotein cholesterol ratio, LAP index lipid accumulation product index, WHt ratio waist-to-height ratio, VA index visceral adiposity index.
